# Genomic islands 1 and 2 play key roles in the evolution of extensively drug-resistant ST235 isolates of *Pseudomonas aeruginosa*

**DOI:** 10.1098/rsob.150175

**Published:** 2016-03-09

**Authors:** Piklu Roy Chowdhury, Martin Scott, Paul Worden, Peter Huntington, Bernard Hudson, Thomas Karagiannis, Ian G. Charles, Steven P. Djordjevic

**Affiliations:** 1 The ithree institute, Faculty of Science, University of Technology, Sydney, PO Box 123, Broadway, New South Wales 2007, Australia; 2 Department of Primary Industries, Elizabeth Macarthur Agriculture Institute, PMB 4008, Camden, New South Wales 2567, Australia; 3 Pathology North, The Royal North Shore Hospital, St Leonards, New South Wales 2065, Australia; 4 SEALS Department of Microbiology, Level 4, Campus Centre Prince of Wales Hospital, Baker Street, Randwick, New South Wales 2031, Australia

**Keywords:** *Pseudomonas aeruginosa*, multiple drug resistance, genomic island, class 1 integron, β**-**lactamases

## Abstract

*Pseudomonas aeruginosa* are noscomially acquired, opportunistic pathogens that pose a major threat to the health of burns patients and the immunocompromised. We sequenced the genomes of *P. aeruginosa* isolates RNS_PA1, RNS_PA46 and RNS_PAE05, which displayed resistance to almost all frontline antibiotics, including gentamicin, piperacillin, timentin, meropenem, ceftazidime and colistin. We provide evidence that the isolates are representatives of *P. aeruginosa* sequence type (ST) 235 and carry Tn*6162* and Tn*6163* in genomic islands 1 (GI1) and 2 (GI2), respectively. GI1 disrupts the *endA* gene at precisely the same chromosomal location as in *P. aeruginosa* strain VR-143/97, of unknown ST, creating an identical CA direct repeat. The class 1 integron associated with Tn*6163* in GI2 carries a *bla*_GES-5_–*aacA4*–*gcuE15*–*aphA15* cassette array conferring resistance to carbapenems and aminoglycosides. GI2 is flanked by a 12 nt direct repeat motif, abuts a tRNA-gly gene, and encodes proteins with putative roles in integration, conjugative transfer as well as integrative conjugative element-specific proteins. This suggests that GI2 may have evolved from a novel integrative conjugative element. Our data provide further support to the hypothesis that genomic islands play an important role in de novo evolution of multiple antibiotic resistance phenotypes in *P. aeruginosa*.

## 1. Introduction

*Pseudomonas aeruginosa* is an opportunistic pathogen and a significant threat to the health of immunocompromised patients hospitalized for periods longer than 7 days [1]. It is a frequent cause of nosocomially derived infections [2,3] and can survive on hospital fomites for extended periods [4–7]. Multiple drug-resistant (MDR), extensively drug-resistant (XDR) and pan-resistant [8,9] *P. aeruginosa* linages cause life-threatening infections in nosocomial environments and severely restrict drug options to treat infection. As such, MDR *P. aeruginosa* is expected to contribute significantly to increased rates of morbidity and mortality in hospital environments in the twenty-first century.

The population structure of *P. aeruginosa* is described as non-clonal and epidemic [10]. Several high-risk clonal lineages including ST111, ST235 and ST175 carry genes that confer resistance to β-lactam antibiotics [10–18] and ST235 clones commonly found in Asia are predominantly associated with the dissemination of IMP type β-lactamase resistance genes [15–17,19–22]. MDR *P. aeruginosa* that encode resistance to the Carbapenem group of antibiotics via the production of molecular class A β-lactamase and class B β-lactamase (metallo-β-lactamases, MBLs) enzymes pose a serious threat to the health of humans and animals because only a limited number of drugs remain available to counteract infections caused by these pathogens. Genes encoding resistance to the molecular class A β-lactamase, extended-spectrum β-lactamase (ESBLs) and MBL antibiotics are often carried on class 1 integrons.

The acquisition of class 1 integrons plays a significant role in the evolution of strains of *P. aeruginosa* that are recalcitrant to combination antibiotic therapy, thereby making them extensively drug-resistant (XDR) [23]. However, class 1 integrons are defective transposons, and an association with functional transposons, genomic islands and plasmids is critical for their dissemination. Within the Enterobacteriaceae, plasmids play a key role in mobilizing class 1 integrons. In *P. aeruginosa*, numerous studies correlate the presence of class 1 integrons with multiple antibiotic resistance, but the genetic context surrounding class 1 integrons is often not investigated. Our earlier studies implicated the important role played by genomic islands in the capture and spread of clustered antibiotic resistance genes within *P. aeruginosa* [24–26].

In 2009, we described a 25.5 kb transposon Tn*6060*, which contained two outwardly facing class 1 integrons encoding resistance to eight antibiotics. Tn*6060* is found in the *res* site of a transposon with more than 99% sequence identity to Tn*1403* within a genomic island (described as GI1) on the chromosome of *P. aeruginosa* strain 37308 [25]. Novel class 1 integrons and transposons were subsequently described in at least four distinct chromosomal locations among clinical isolates from Australia and Uruguay [26]. These integrons carried identical cassette arrays comprising *aadA6* (encoding resistance to streptomycin and spectinomycin) and *gcuD* (formerly orfD) within a novel transposon, Tn*6162,* that is located at precisely the same position as Tn*6060* in GI1. Class 1 integrons harbouring the *aadA6*-*gcuD* array have been reported from geographically divergent regions including France [27] and in an extensively drug-resistant *P. aeruginosa* isolate from Thailand [28], but the genetic context of the integron-arrays remains unknown. We also described an Australian *P. aeruginosa* strain, C79, containing a second-class 1 integron that carries *bla*_GES5_ (encoding resistance to extended-spectrum β-lactams), an *aacA4*-like gene cassette (encoding resistance to aminoglycosides), *gcuE15* (unknown function), *aphA15* (aminoglycoside resistance) and several IS elements in genomic island 2 (GI2) [26]. The integron in strain C79 was found within a mercury resistance transposon Tn*4380* known as Tn*6163,* also located in a genomic island, described as island 2 or GI2 [26]. While Tn*6163* was not detected in isolates from Uruguay, it was identified in seven clonally related clinical isolates of *P. aeruginosa* collected from Sydney in 2010 [26]. Although the structures of transposons Tn*6060*, Tn*6162* and Tn*6163* have been described in detail, the genomic islands harbouring them were not characterized in any of our previous studies.

In *P. aeruginosa* strain VR-143/97, the *bla*_VIM-1_ metallo-β-lactamase gene cassette is associated with a class 1 integron (In70.2) in a defective Tn*402*-like transposon that is inserted in the *urf2* gene of a Tn*3-*derivate transposon known as Tn*6249*. Like Tn*6060*, Tn*6249* has a complex structure and carries two class 1 integrons (In90 and In70.2) in a tail-to-tail orientation [29]. Tn*6249* is located in a PACS171b-like genomic island, described earlier as GI1 in relation to Tn*6060* [25]*,* within the *endA* gene of the *P. aeruginosa* chromosome [29]. Tn*6249* shares structural identity with Tn*6060,* although the gene cassettes carried by In90 are different and a part of the Tn*3* backbone is missing in Tn*6249* [25]. Notably, Tn*6162* is also located at an identical position in the genomic backbone of GI1 as Tn*6060,* but has a simpler complex resistance locus (CRL) consisting of one class 1 integron. Consequently, it has been hypothesized that insertion of a Tn*6162*-like ancestor into the PACS171b-like genomic island (VR-143/97; 51 424 nt) preceded the genetic events that gave rise to Tn*6249* and Tn*6060* in clonally dispersed lineages of *P. aeruginosa* [29].

Here we present an in-depth, whole-genome-based analysis of three MDR ST235 isolates carrying both Tn*6162* in GI1 and Tn*6163* in GI2. All three isolates are resistant to the entire range of antibiotics used to treat *P. aeruginosa* infections except colistin (intermediate resistance to colistin) and are part of a larger collection of phylogenetically related MDR isolates recovered in 2006 and 2007 from a burns ward in a hospital in Sydney. The genetic structure of GI1 and GI2 found in *P. aeruginosa* isolates in Sydney is presented. Our study highlights the roles played by GI1 and GI2 in the capture and dissemination of multiple antibiotic resistance genes in *P. aeruginosa* ST235 in the Sydney basin.

## 2. Material and methods

### 2.1. Strains, isolation and culture conditions

The *P. aeruginosa* isolates examined in this study were obtained during an outbreak of a multiple antibiotic-resistant *P. aeruginosa* infection at the Royal North Shore Hospital (RNSH) in the burns unit during 2006 and 2007. Isolates RNS_PA1 (rectal swab, November 2006), RNS_PA46 (burn wound, May 2007) and RNS_PAE05 (hand sanitizer, April 2007) were recovered as part of routine microbiological surveillance during the outbreak. The isolates were identified as *P. aeruginosa* using standard microbiological protocols [30] and stored at −80°C.

For molecular studies, the isolates were routinely grown at 37°C on Luria–Bertani (LB) agar or overnight in LB broth with shaking at 125 r.p.m. For antibiotic sensitivity testing, cells from two to three colonies growing on blood agar were subcultured in tryptone soy broth (TSB) for 3 h at 37°C prior to plating on antibiotic supplemented Columbia agar. For extraction of sequencing quality DNA, cells were grown in 5 ml of M63 minimal salts broth for 20 h at 37°C in a shaking incubator set at 125 r.p.m., harvested by centrifugation and stored at −80°C.

### 2.2. DNA extraction

Genomic DNA for PCR was extracted from 200 µl of a TSB culture inoculated with several colonies from blood agar plates. DNA was extracted on a BioRobot M48 workstation (Qiagen) at RNSH following the manufacturer's instructions. For next-generation genomic sequencing, DNA samples were extracted using Bioline's ISOLATE-II Genomic DNA extraction kit following recommended protocols.

### 2.3. RAPD analysis

RAPD analysis was conducted using puRe Taq Ready-To-Go PCR beads (GE Healthcare) with 20 ng of template DNA, using published protocols [31]. The amplicons were resolved and visualized on a QIAxcel (Qiagen) 12-channel capillary electrophoresis system. Data were exported to BioNumerics 6 (Applied Maths) software for comparison and analysis.

### 2.4. PCR

Routine PCR was conducted in a total volume of 20 µl using Mango Taq (Bioline) master mix. Cycling conditions comprised an initial denaturation step at 94°C for 2 min 30 s, followed by 30 cycles of denaturation (94°C for 30 s) annealing (60°C for 30 s) and extension (72°C for 5 min). An EmeraldAmp Taq polymerase (Master Mix) was used in long-range PCR to bridge Illumina sequence scaffolds. Primer sequences and amplicon sizes are listed in electronic supplementary material, table S1.

### 2.5. Antibiotic resistance testing

Antibiotic resistance profiles were generated following the CLSI guidelines [32]. Plates were incubated overnight at 37°C. The antibiotic concentration at which colony growth was inhibited was noted as the minimum inhibitory concentration (MIC). Reference strains used in the experiment were *P. aeruginosa* ATCC27853 and *E. coli* ATCC25922.

### 2.6. Sanger sequencing and whole genome sequencing

Promega Wizard SV PCR and Gel Clean Up kits were used to prepare PCR amplicons for sequencing. Sanger sequencing was performed at the Australian Genome Research Facility at Westmead Hospital, Westmead, NSW.

Genome sequencing was performed using a bench top Illumina MiSeq sequencer and MiSeq V3 chemistry at the ithree institute at UTS [33]. Genomes were submitted to GenBank using the following accession numbers: RNS_PA1 (SAMN04038435), RNS_PA46 (SAMN04038437) and RNS_PAE05 (SAMN04038440). 400 nt Illumina reads were assembled using the A5-MiSeq (ngopt_a5pipeline_linux-x64_20130919)) de novo assembly pipeline [34]. Preliminary genome annotations were performed with an online version of RAST [35,36] using FigFAM release 70. Sequences of interest from the preliminary annotation were manually curated using iterative NCBI-ORF finder software coupled with BLASTn and BLASTp analysis. ORFs that returned identity across 100% of the input query sequence in BLASTp analysis were scored.

### 2.7. Bioinformatic analysis

Phylogenetic comparisons of the three *P. aeruginosa* genomes (RNS_PA1, RNS_PA46 and RNS_PAE05) with nine completely closed *P. aeruginosa* genomes available in NCBI (accessed on 3 September 2015) was performed using PhyloSift [37]. Genome sequences of *Vibrio cholerae* N16961 (NC_002505.1) and *Salmonella enterica* serovar *typhimurium* DT104 (NC_022569.1) were used as outgroups in the analysis. FigTree v. 1.4.0 (http://tree.bio.ed.ac.uk/software/figtree/) was used to draw the phylogenetic tree. Two closely related, finished genomes (PA_NCGM2 and PA_PA14) were chosen from the PhyloSift output and used to do a second phylogenetic analysis based on the whole genome alignment protocol in REALPHY [38].

The MLST database (http://pubmlst.org/paeruginosa/) was used to identify the sequence type (ST) of the isolates. Whole genome comparative analysis was performed using MAUVE [39], and regions of interest between the three genomes were further characterized using iterative BLASTn and BLASTp searches [40]. Figures were compiled using BRIG [41].

## 3. Results

### 3.1. Characterization of three multiple drug-resistant *Pseudomonas aeruginosa* isolates from the Royal North Shore Hospital burns unit

The three MDR *P. aeruginosa* isolates examined in this study were representative of a collection of 36 isolates from RNSH that shared 90% similarity by RAPD analyses (electronic supplementary material, figure S1). PCR analyses indicated that all 36 isolates carried a class 1 integron and a characteristic 1800 bp cassette array amplicon akin to the array present in Tn*6162*. The amplicon was shown to contain *aadA6* and *gcuD* gene cassettes by nested PCR using a primer pair that internally annealed to the *aadA6* (HS513) and *gcuD* (gcuD-Rv) genes, confirming that these isolates carry Tn*6162*. Of the 36 isolates, 27 also produced a 2407 bp amplicon using a forward primer (HS1299) in *bla*_GES-5_ and reverse primer (HS1236) in *aphA15*. The 2407 bp amplicon is indicative of the presence of Tn*6163* containing *bla*_GES-5_–*aacA4*–*gcuE15*–*aphA15* gene cassettes [26]. Isolates included in this study were tested for sensitivity to antibiotics used at RNSH to treat *P. aeruginosa* infections, including gentamicin, piperacillin, ticarcillin and clavulanic acid, meropenem, ceftazidime and colistin (table 1). Defining features of the three isolates selected for genome sequence analysis were (i) that they were isolated from different patients/sources at the RNSH over a seventh month period, (ii) that they displayed minor differences in their resistance profile and intermediate resistance to colistin (table 1), a last line treatment option for *P. aeruginosa* infections, and (iii) a suspicion that they carried Tn*6162* and Tn*6163* using the PCR strategy described above. As such, these three isolates represent a set that predates (by 4 years) strains from which Tn*6162* and Tn*6163* were originally described in Australia [26] from a different hospital in the Sydney basin.

**Table 1.** Antibiotic resistance profiles of *P. aeruginosa* isolates. R, resistant to antibiotic; I, intermediate resistance to antibiotic; S, susceptible to antibiotic; number in parentheses is the MIC in µl ml^−1^. GEN, gentamicin (aminoglycoside); PIP, piperacillin (extended-spectrum β-lactam); TCA, ticracillin [β-lactam]/clavulanic acid [β-lactamase inhibitor]; MPM, meropenem (carbapenem); CAZ, ceftazidime (third-generation cephalosporin); COL, colistin (polymixin E).

isolate	GEN^a^	PIP^b^	TCA^c^	MPM^d^	CAZ^e^	COL^f^
RNS_PA1	R (>32)	R (>256)	R (128/2)	R (64)	R (32)	I (4)
RNS_PA46	R (>32)	R (128)	R (128/2)	R (128)	R (32)	I (4)
RNS_PAE05	R (>32)	R (128)	R (128/2)	R (128)	R (32)	I (4)

^a^S ≤ 4, I = 8, R ≥ 16.

^b^S ≤ 64, R ≥ 128.

^c^S ≤ 64/2, R ≥ 128/2.

^d^S ≤ 4, I = 8, R ≥ 16.

^e^S ≤ 8, I = 16, R ≥ 32.

^f^S ≤ 2, I = 4, R ≥ 8.

### 3.2. Phylogenetic analysis of the three isolates

**Figure 1.**
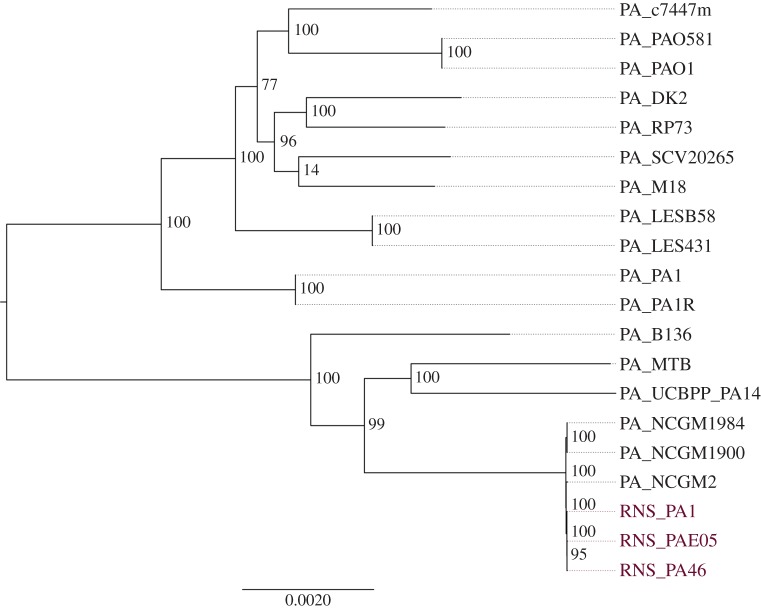
Phylogenetic tree based on whole genome alignments created using REALPHY.

Raw reads were assembled (assembly statistics in electronic supplementary material, table S2) using the A5 pipeline [34] yielding approximately 170 scaffolds with an average of 35–38 × coverage and N50 scores from 99 078 to 111 819 nt. The three *P. aeruginosa* genomes (RNS_PA1, RNS_PA46 and RNS_PAE05) were compared with 18 finished *Pseudomonas* genomes deposited in GenBank using PhyloSift. Two of the three isolates (RNS_PA1 and RNS_PA46) clustered in a single clade with *P. aeruginosa* isolate NCGM2 (electronic supplementary material, figure S2). PhyloSift infers phylogenetic relationships within bacterial populations based on the amino acid sequence identity of 37 conserved core marker genes [37]. The branch that clustered RNS_PA1, RNS_PA46 with NCGM2 displayed a confidence value of 0.92, indicating that *P. aeruginosa* NCGM2 is the appropriate reference genome for comparative genomic analyses. Isolate RNS_PAE05, from the hand sanitizer bottle, resided on a separate clade. eMLST analysis [42] of NCGM2 and all three of our isolates showed that they belong to ST235. *P. aeruginosa* isolate PA14 resides in a clade that shares a common ancestral node with RNS_PA1, RNS_PA46 and NCGM2. PA14 belongs to the ST253 clade and is a multiple antibiotic-resistant clinical isolate implicated in a wide variety of diseases [43]. All 20 isolates branching out of PA_PA7 were used to set up a whole genome alignment-based phylogenetic analysis using REALPHY [38]. NCGM2 and PA14 were used as reference genomes in this analysis. The tree generated by aligning whole genomes places RNS_PA1, RNS_PA46 and RNS_PAE05 in a single clade (bootstrap value of 100) and strain NCGM2 as the closest neighbour (figure 1).

### 3.3. Characterization of genomic islands GI1, GI2 and *exoU* in *Pseudomonas aeruginosa* isolate RNS_PA1

**Figure 2.**
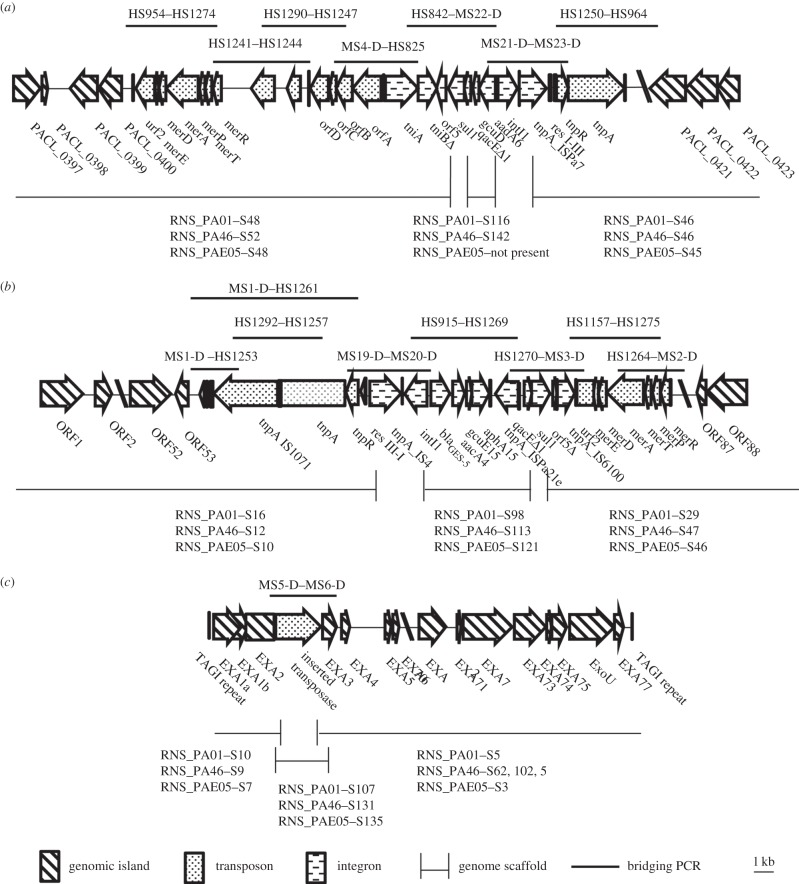
The PCRs used to link scaffold breaks within the three genomic islands found in isolate RNS_PA1. (*a*) Tn6162 in GI1, (*b*) Tn6163 in GI2 and (*c*) *exoU* island with identified insertion element. PCRs shown with primers above the structure and the scaffolds for MS1 shown below each structure. Amplicons were verified using Sanger sequencing.

Scaffolds spanning GI1 and GI2 in *P. aeruginosa* RNS_PA1 were assembled by targeted PCR and Sanger sequencing followed by BLASTn alignments with published sequences. Transposons structures were confirmed using overlapping PCR experiments (figure 2*a* and 2*b*, respectively).

**Figure 3.**
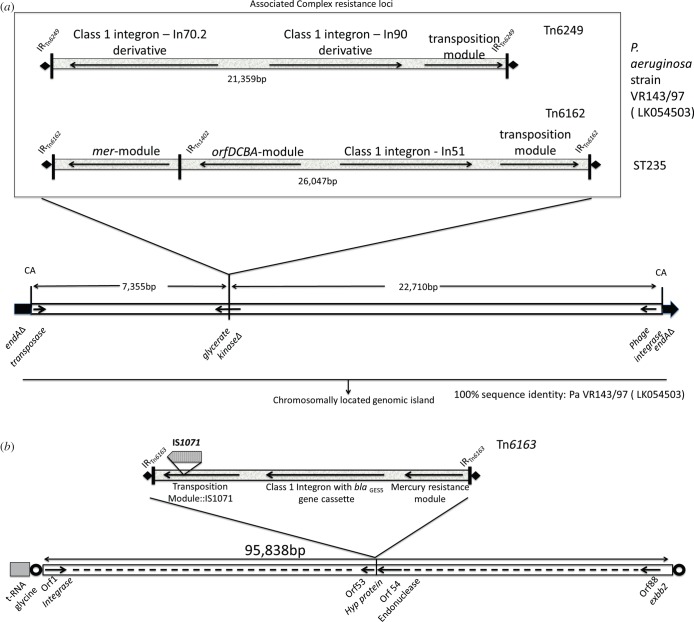
Diagrammatic representation of the precise location of the complex resistance loci on the respective genomic islands. (*a*) Comparison of the complex resistance loci in Genomic Island 1 in *P. aeruginosa* strain VR-143/97 and in globally disseminated ST235, and their location in the glycerate kinase gene of GI1. (*b*) Complex resistance region of Genomic Island 2, which includes the precise location of Tn*6163*. Open circles represent 12 bp direct repeat (AACGCCAGGGAA) generated due to insertion of GI2. Bold diamond depicts 5 bp direct repeat (CTCAA) generated owing to insertion Tn*6163*.

GI1 in RNS_PA1 comprises a 54 840 nt-long DNA segment and harbours Tn*6162*. GI1 was originally described as part of a genomic fragment in clone fa1389 (GenBank accession: EU595750) from *P. aeruginosa* isolate PACs171b and was thought to be restricted to isolates from patients with cystic fibrosis [44]. An island that contains significant portions of the fa1389 clone was recently found inserted in the chromosomally located *endA* gene in strain VR-143/97 isolated from Italy in 1997. We also identified regions within the fa1389 clone in isolates sourced from patients without cystic fibrosis in Australia and South America [25,26]. Insertion of the island creates a signature 2 bp (CA) direct repeat (figure 3*a*) at the chromosomal insertion site. Tn*6162* in our three isolates is 26 056 nt long and has 99% sequence identity over 100% of the query sequence to the first described sequence of Tn*6162* (JF826498.1). Our sequence contains five single nucleotide polymorphisms compared with Tn*6162* described previously. Beyond the CRL, the sequence of the genomic island is identical at both ends to the GI sequence from strain VR-143/97 (figure 3*a*).

GI2, which carries Tn*6163*, was identified on three scaffolds. Gap closing PCRs followed by Sanger sequencing led to the reconstruction of the GI2 sequence (figure 3*b*; electronic supplementary material, figure S4). In isolate RNS_PA1, GI2 (115 604 nt) comprises 94 368 nt of chromosomal backbone (average G+C content 63.5%) and is located adjacent to a tRNA-gly gene. Tn*6163* comprises 21 236 nt and displays 99% sequence identity to Tn*6163* (GenBank accession JF826499) with 15 SNPs and eight gaps, and is flanked by the characteristic 5 nt direct CTCAA repeats (figure 3*b*). A complete integrase gene (WP_003158602.1) resides next to the tRNA insertion site. Manual annotation of the GI2 backbone identified 88 ORFs (electronic supplementary material, figure S4). Details of the gene content in GI2 are presented in electronic supplementary material, table S3. A 12 nt direct repeat with the sequence AACGCCAGGGAA flanks the ends of GI2, suggesting that it is likely to be a recent insertion event at this site. Genes encoding proteins with roles in integration (integrase), conjugative transfer as well as integrative conjugative element-specific proteins are present, indicating that GI2 may be a novel integrative conjugative element.

BLAST analysis of the entire GI2 backbone identified a match (99% sequence identity) to a region in *P. aeruginosa* strain NCGM 1900 (AP104622) suggesting that this strain contains a variant of GI2 (figure 3). Further analysis of the matching sequence in NCGM 1900 failed to identify any evidence of Tn*6163*. BLAST alignments also identified a fragment (99% sequence identity) comprising 26 659 nt from ORF63 to the end of GI2 in the NCGM2 genome (NC_017549) indicating the island, like GI1, is not restricted within *P. aeruginosa* isolates collected in Australia.

The *exoU* genomic island in RNS_PA1 which harbours the gene encoding the potent cytotoxin ExoU is 83 830 nt in length and shows 99% sequence identity to the *exoU* island A in GenBank entry DQ437742 (81 751 nt). The sequence in RNS_PA1 differs only by the presence of a 2059 nt insertion element (figure 2*c*). The type III secretion system (T3SS) in *P. aeruginosa* plays an important role in pathogenicity by transporting effector molecules such as the cytotoxin ExoU into host cells. The structural components of the T3SS are located on a 23.9 kb region in the PA14 genome and these were aligned to corresponding regions of the three genomes in our study (electronic supplementary material, figure S3). RNS_PA1 had 140 SNPs and seven gaps, RNS_PA46 had 134 SNPs and one gap, and NCGM2 had 140 SNPs and seven gaps, when compared with the T3SS in PA14. The T3SS in isolate RNS_PAE05 had 138 SNPs, one gap and a 52 bp deletion located within a regulatory gene *exsC*. In *Vibrio parahemolyticus*, deletion of the *exsC* gene has been shown to shut down the expression of T3SS [45].

### 3.4. Comparative genome analysis

**Figure 4.**
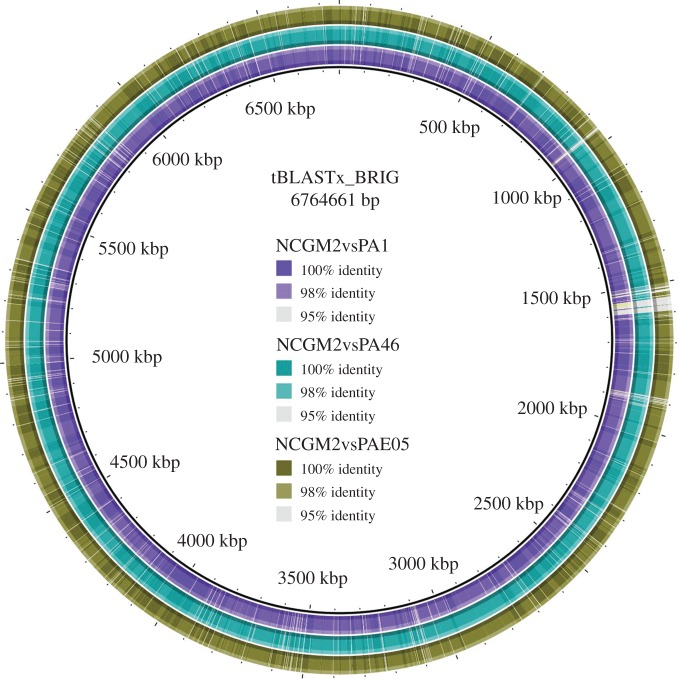
Comparative tBLASTx analysis of the three *P. aeruginosa* genomes against the reference NCGM2 genome.

All three genomes were tiled against the closed genome sequence of *P. aeruginosa* NCGM2. The genome of strain NCGM2 comprises 6 764 661 nt, and is slightly smaller than the genomes from RNS_PA1, RNS_PA46 and RNS_PAE05. tBLASTx analysis comparing whole genomes of RNS_PA1, RNS_PA46 and RNS_PAE05 independently with NCGM2 (figure 4) identified a major region of difference close to the 1500 kbp marker. Bi-directional BLASTp analysis comparing each of our isolates with NCGM2 identified four regions of difference containing phage-derived proteins. These analyses suggest that ST235 isolates derived from different geographical regions have likely been infected with different phage populations and have been subjected to novel phage-mediated genomic rearrangements.

**Figure 5.**
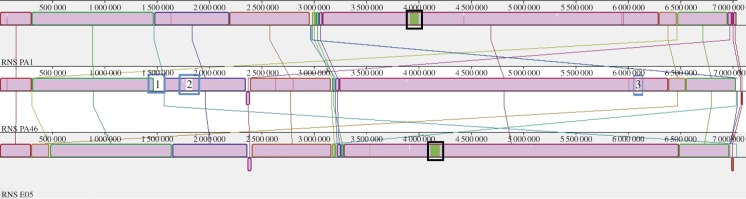
Mauve alignments of the RNS_PA1, RNS_PA46 and RNS_PAE05. Black boxes around the green segment in genome RNS_PA1 and RNS_PAE05 represent parts of scaffold 4 and 8 in the genome sequence, respectively. Blue box 1 in genome RNS_PA46 represents scaffold 27, box 2 represents scaffold 40 and box 3 represents scaffold 77.

Progressive MAUVE alignment of the three genomes was used to identify scaffolds that define the core and accessory genomes in RNS_PA1, RNS_PA46 and RNS_PAE05 (figure 5). Regions coloured in magenta represent shared sequence identity in RNS_PA1, RNS_PA46 and RNS_PAE05. Blue-boxed regions in the core genome of RNS_PA46 are unique to this strain. Scaffold 27 (blue box 1) from strain RNS_PA46 contains genes that encode proteins relating to a ‘mobile genetic element’ like integrase, conjugation proteins and a series of uncharacterized hypothetical proteins. Scaffolds 40 and 77 (boxes 2 and 3, respectively, in figure 5) carry genes encoding phage-associated proteins. Electronic supplementary material, table S4 provides a description of the gene content of the unaligned scaffolds generated by preliminary RAST annotation of regions greater than 1 kb.

In summary, the differences noted in the genomes of RNS_PA1, RNS_PA46 and RNS_PAE05 appear to be phage-related and other laterally acquired regions. RNS_PA46 has the biggest genome, comprising 6672 ORFs, and RNS_PA1 is the smallest genome, with 6564 predicted ORFs. Table 2 shows a summary of SNPs and indels identified at least 100 nt from a scaffold break. RNS_PAE05 contains the largest number of SNPs of the three genomes. These data show that of the functionally defined genes, major SNP differences in the genomes of RNS_PA1, RNS_PA46 and RNS_PAE05 were localized within housekeeping genes. For example, the DNA polymerase gene *dnaX*, glucose carbohydrate outer membrane porin gene *oprB* and riboflavin-specific deaminase reductases gene *ribD* exhibited SNPs. However, differences were also identified in a number of other genes that were annotated as hypothetical proteins by RAST.

**Table 2.** Comparison of SNPs identified in *P. aeruginosa* isolates included in this study.

	SNP			indel	
	individual SNPs	clustered SNP groups	genes/ORFs with clustered SNPs	indels present	large indels^a^
RNS_PA1	101	2	61 glucose/carbohydrate outer membrane porin genes 18 riboflavin-specific deaminase/reductases genes	47	0
RNS_PA46	119	4	34 hypothetical protein 13 putative MFS transporter 10 hypothetical protein 19 hypothetical protein (NCGM2_4844)	49	6
RNS_PAE05	308	11	27 hypothetical protein 18 putative glycerol kinase 96 dnaX 10 isochorismatase family hydrolase 15 putative transmembrane sensor 13 hypothetical protein 15 no ORF 18 no ORF 16 hypothetical protein 18 elongation factor Tu 20 putative gamma-glutamyltranspeptidase precursor	45	0

^a^Large indels defined as larger than 5 kb.

### 3.5. Genomewide identification of resistance and virulence genes

RNS_PA1, RNS_PA46 and RNS_PAE05 carried identical variants of all the resistance and virulence genes listed in table 3. In addition, we also surveyed the Comprehensive Antibiotic Resistance Database (http://arpcard.mcmaster.ca) with our genome sequences (electronic supplementary material, table S5). The repertoire of antibiotic resistance genes identified in each of the strains is likely to account for their resistance phenotype. Mutations in *phoQ* and *pmrB* are known to have a role in colistin resistance [46]. In the genomes of RNS_PA1, RNS_PA46 and RNS_PAE05, an F76Y mutation in *phoQ* was identified and is unique to our isolates. We also identified a V15I mutation in *pmrB*. This same mutation was recently reported to alter colistin sensitivity in *P. aeruginosa* strain P165 [47]. We speculate that these mutations have probably contributed to the intermediate colistin resistance phenotype exhibited in our three Sydney isolates. While the genome of NCGM2 carries a different suite of resistance genes to the ones in RNS_PA1, RNS_PA46 and RNS_PAE05, we cannot comment any further as the resistance profile of NCGM2 has not been reported.

**Table 3.** Antibiotic resistance and virulence genes in *P. aeruginosa* isolates.

antibiotic resistance genes	RNS_PA1	RNS_PA46	RNS_PAE05	NCGM2	NCGM 1900
*aadA6* (encodes resistance to aminoglycoside) (JF826498.1: 24 514-25 359)	present 100% nBLAST	present 100% nBLAST	present 100% nBLAST		present 100% nBLAST
*aphA15* (encodes resistance to aminoglycosides) (JF826499.1: 13 868-14 662)	present 100% nBLAST	present 100% nBLAST	present 100% nBLAST		
*nfxB* (encodes resistance to norfloxacin) (CP000438.1: 5 428 030–5 428 593)	present 98% nBLAST 100% pBLAST	present 98% nBLAST 100% pBLAST	present 98% nBLAST 100% pBLAST	present 98% nBLAST 100% pBLAST	present 98% nBLAST 100% pBLAST
*bla_GES-5_* (encodes resistance to β-lactam) (JF826499.1: 11 979–12 842)	present 100% nBLAST	present 100% nBLAST	present 100% nBLAST		
*ampC* (β-lactam) (CP000438.1: 932 631–933 824)	present 100% nBLAST	present 100% nBLAST	present 100% nBLAST	present 100% nBLAST	present 99% nBLAST 100% pBLAST
*phoQ* (mutations known to cause colistin resistance) (AE004091.2: 1 278 362–1 279 708)	present 99% nBLAST 99% pBLAST F76Y	present 99% nBLAST 99% pBLAST F76Y	present 99% nBLAST 99% pBLAST F76Y	present 99% nBLAST 99% pBLAST F76Y	
*pmrB* (mutations known to cause colistin resistance) (AE004091.2|:5 364 760–5 366 193)	present 99% nBLAST 99% pBLAST V15I	present 99% nBLAST 99% pBLAST V15I	present 99% nBLAST 99% pBLAST V15I		

putative virulence genes	RNS_PA1	RNS_PA46	RNS_PAE05	NCGM2	NCGM 1900
*exoT* (effector of T3SS) (AE004091.2: 58786–60 159)	present 99% nBLAST 100% pBLAST	present 99% nBLAST 100% pBLAST	present 99% nBLAST 100% pBLAST	present 99% nBLAST 100% pBLAST	present 99% nBLAST 100% pBLAT
*exoY* (effector of T3SS) (AE004091.2: 2 410 344–2 411 480)	present non-functional 99% nBLAST stop codon present in middle of sequence	present non-functional 99% nBLAST stop codon present in middle of sequence	present non-functional 99% nBLAST stop codon present in middle of sequence	present non-functional 99% nBLAST stop codon present in middle of sequence	
*aprA* (extracellular protease) (AE004091.2: 1 355 631–1 357 070)	present 99% nBLAST 100% pBLAST	present 99% nBLAST 100% pBLAST	present 99% nBLAST 100% pBLAST	present 99% nBLAST 100% pBLAST	present 99% nBLAST 100% pBLAST
*lasA* (extracellular protease) (AE004091.2: 2 410 344–2 411 480)	present 98% nBLAST 100% pBLAST	present 98% nBLAST 100% PBLAST	present 98% nBLAST 100% pBLAST	present 98% nBLAST 100% pBLAST	
*lasB* (extracellular protease) (AE004091.2: 4 168 987–4 170 483)	present 98% nBLAST 100% pBLAST	present 98% nBLAST 100% pBLAST	present 98% nBLAST 100% pBLAST	present 98% nBLAST 100% pBLAST	present 98% nBLAST 100% pBLAST
*exoU* (acute cytotoxin) (CP000438.1(PA14): 4 580 957–4 583 020)	present 99% nBLAST 100% pBLAST	present 99% nBLAST 100% pBLAST	present 99% nBLAST 100% pBLAST	present 99% nBLAST 100% pBLAST	present 99% nBLAST 100% pBLAST
*ladS* (promotes biofilm formation) (AE004091.2: 4 453 289–4 455 676)	present 99% nBLAST 100% pBLAST	present 99% nBLAST 100% pBLAST	present 99% nBLAST 100% pBLAST	present 99% nBLAST 100% pBLAST	present 99% nBLAST 100% pBLAST

## 4. Discussion

Previously we showed that two genomic islands are implicated in mobilizing transposons that carry multiple antibiotic resistance genes in clinical isolates of *P. aeruginosa* [26,48]. Here, we describe the genetic architecture of two genomic islands implicated in the dissemination of multiple antibiotic resistance genes in three *P. aeruginosa* ST235 isolates from Sydney and provide further evidence supporting our hypothesis that genomic islands are major players in the dissemination of multiple antibiotic resistance. We have characterized GI1 in three Sydney isolates which are representatives of ST235, and the structure is very similar to the one described recently from isolate VR-143/97 from Italy. In addition, we define, for the first time, both the boundaries of and the genetic composition of genomic island 2. GI2 was originally proposed to be restricted to isolates collected from the Sydney basin. However, in this study, we provide evidence that suggests that GI2 is not restricted to isolates from a specific geographical location and may be a characteristic of the ST235 clonal lineage.

In our Sydney isolates, we show that GI1 carries Tn*6162* (figure 3) and has inserted into the *endA* gene at precisely the same position as a PACS171b-like genomic island in multiple antibiotic-resistant *P. aeruginosa* isolate VR-143/97 isolated in Italy in 1997 [45]. The PACS171b-like genomic island in strain VR-143/97 harbours Tn*6249*, a complex transposon carrying two different class 1 integrons, one of which houses a VIM-1 gene cassette. The genome sequence for strain VR-143/97 has not been released and as such we cannot determine if VR-143/97 belongs to the ST235 lineage. In Australian isolates of *P. aeruginosa*, GI2 harbours Tn*6163*. Tn*6163* contains a class 1 integron with *bla*_GES-5_–*aacA4*–*gcuE15*–*aphA15* gene cassettes. It is not known if strain VR-143/97 also harbours Tn*6163*. Notably, BLAST analysis of the entire GI2 backbone identified (99% sequence identity) a region in ST235 *P. aeruginosa* strain NCGM 1900 raising the possibility that GI2 may be widely disseminated in the ST235 lineage. While GI2 comprises certain features typical of ICE elements described in other bacteria, it has clearly undergone genetic rearrangements in the three genomes described in this study. Currently, we do not have any evidence to indicate GI2 has the capacity to excise from the genome and transfer elsewhere via conjugation.

The three Australian ST235 isolates carry both GI1 and GI2 and display resistance to almost all frontline antibiotics used to control infections caused by *P. aureginosa* at RNSH. Tn*6163* harbours a class 1 integron that contains the Ambler class A β-lactamase gene cassette *bla*_GES-5_ that confers resistance to all β-lactams including carbapenems. It also contains *aphA15* that confers resistance to kanamycin and neomycin. The aminoglycoside resistance gene *aadA6* that confers resistance to gentamicin forms the first cassette in the array of the class 1 integron in Tn*6162*.

GI1 appears to have been integrated into the genome by a phage-integrase-mediated site-specific recombination event. The PHAST database was interrogated to identify phage genes that may facilitate movement of the genomic islands (data not shown), but none were identified. However, given the number of phage-related genetic signatures in all three *P. aeruginosa* genomes, it is possible that parts of GI1 were originally transferred into *P. aeruginosa* by a phage which has subsequently lost function. The *exoU* island A in our Australian isolates had 99% sequence identity to the *exoU* island present in *P. aeruginosa* strains NCGM1900 and NCGM2 from Japan [49]. We identified a novel insertion sequence in *exoU* island A that is unique to the Australian isolates of *P. aeruginosa* described in this study. The closest match to the insertion sequence is found in the genome of *Pseudomonas putida* strain S12 (CP009974). Further work is needed to determine if the insertion sequence can be used as a marker to identify the *exoU* island A variant.

RNS_PA1, RNS_PA46 and RNS_PAE05 carry a comprehensive repertoire of putative virulence genes. In *P. aeruginosa*, the T3SS has four known effectors: ExoU, ExoY, ExoT and ExoS. While clinical isolates have never been shown to carry all four effectors, ExoT has been identified frequently, suggesting it performs a function in pathogenesis [50]. ExoT is known to inhibit host-cell division by interrupting cytokinesis in mammalian cells [50]. In an epithelial cell model, ExoY has adenlylate cyclase activity that leads to increases in intracellular cAMP levels and inhibits cellular invasion [51]. ExoU is an important virulence factor that mediates cell death in macrophages, epithelial cells and fibroblasts [52]. ExoU functions in concert with its chaperone SpcU, and we identified *spcU* in the exoU island A in the genome sequences of all three Sydney isolates. The T3SS effector proteins play a significant role in causing severe pneumonia [53]. LasA and LasB possess elastolytic activity [54], whereas LadS activates a cascade that decreases cytotoxicity and increases biofilm formation [55]. Finally, AprA encodes an alkaline protease that is secreted by the type 1 secretion system [56].

In conclusion, all three Sydney ST235 isolates have the *bla*_GES-5_ gene inserted as a gene cassette within a class 1 integron that forms part of Tn*6163* located in GI2*.* The genomic data presented here and elsewhere (AP014622, AP014646 and [29]) show that genomic islands are playing a seminal role in the dissemination of resistance to a wide range of clinically important antibiotics, including metallo-β-lactams and extended-spectrum β-lactams. It is not known if GI1 and GI2 can be mobilized to other Gram-negative bacteria.

## Supplementary Material

Supplementary Data Tables

## Supplementary Material

Supplementary Figures

## Data accessibility

Genome sequences described in this manuscript were submitted in GenBank and accession numbers are provided in the Methods section.
