# Supplementary material for: Genomic islands 1 and 2 play key roles in the evolution of extensively drug-resistant ST235 isolates of Pseudomonas aeruginosa
Source: Open Biol. 2016 Mar 9;6(3):150175. doi: 10.1098/rsob.150175 (PMC4821235; doi:10.1098/rsob.150175)
Supplement: Supplementary Data Tables [file rsob150175supp1.docx]

Table S1. Primers used in this study

| Primers | Sequence | | PCR | Target | Amplicon | Reference |
| --- | --- | --- | --- | --- | --- | --- |
| Screening Primers | | | | | | |
| HS915 | CGTGCCGTGATCGAAATCCAG | | *intI1* | *intI1* | 330 | [1] |
| HS916 | TTCGTGCCTTCATCCGTTTCC | |  | *intI1* |  |  |
| HS549 | ACTAAGCTTGCCCCTTCCGC | | 3’-CS | *sul1* | 1059 | [1] |
| HS550 | CTAGGCATGATCTAACCCTCGG | |  | *sul1* |  |  |
| HS513 | ATTTGTACGGCTCYGCASTGG | | Internal PCR for the Tn*6162* Cassette array | *aadA6*  *gcuD* | 1442 |  |
| gcuD Rv | CCAGAAGCCAAACGGATAA | |  |  |  |  |
| HS1152 | CGGTCGAGTCAAAAGCTAGG | | GI1 targeting PCR | PACS171b : PACL_0400  PACS171b : PACL_0400 | 636 | [2] |
| HS1153 | CAGTACCAACCCCCTCAAAA | |  |  |  |  |
| HS1236 | TCGCCATGTGTGACTACCAG | | Internal PCR for the Tn*6163* cassette array | *aphA15* | 2407 |  |
| HS1299 | TGCGTCGGAAAAATTAACCT | |  | *bla_GES-5_* |  |  |
| HS1231 | AGCCGTCCTTGATGAACAT | | GI2 targeting PCR |  |  | [2] |
| HS1232 | ATTATCCTTTCCGCGCTTCT | |  |  |  |  |
|  | | **Tn*6162* and Tn*6163* Confirmatory Primers** | | | | |
| HS954 | CAACCACGTAAGCAACTG | |  | Before PACL_0401 | 4791 |  |
| HS1274 | AGTTCGGATAGCACGGTTTC | |  | *merR* Tn*6162* |  |  |
| HS1241 | AACCGTGCTATCCGAACTTG | |  | *merR* Tn*6162* | 4903 |  |
| HS1244 | ACAGGGACTTGATGGCTTTG | |  | Between *orfs* and res sites |  |  |
| HS1290 | TGTTGCTGATCGACTTGCTT | |  | Between *mer* and *orfs* | 4182 |  |
| HS1247 | TCGGAGCGGTGAAATAGAAC | |  | *orfB* Tn*6162* |  |  |
| MS04-D | GCTTTGATCCAGCTCTACCG | |  | *orfC* | 3975 | This study |
| HS825 | TGTTTTCGGAATCGTAGTCGC | |  | *tniA* |  |  |
| HS842 | ATGCTCAATACTCGTGTGCACC | |  | *tniA* | 4839 |  |
| HS1226 | GCTGGGTTTGCCGTTTCTCT | |  | *gcuD* |  |  |
| HS1250 | GTGTGCGCATCGAGTTCTT | |  | *tnpR* Tn*6162* | 3489 |  |
| HS964 | GAAGGGAGGTTGGATGAA | |  | PACL_0401 |  |  |
| MS01-D | ATGCGGTCTTGAACGTATCC | |  |  | 1459 | This study |
| HS1253 | AAGCATTGGGATAGTCTGGTG | |  | IS*1071* |  |  |
| HS1292 | GCATTTCATCGACACGTTTG | |  | IS*1071* | 3198 |  |
| HS1257 | CCAAGCTCTACATCCCGAAG | |  | *tnpA* Tn*6163* |  |  |
| HS915 | CGTGCCGTGATCGAAATCCAG | |  | *intI1* | 4568 |  |
| HS1269 | TGGCGTTATGTACAGGAGCA | |  | *tnpA* IS*Pa21* |  |  |
| HS1270 | CAGGCTTCCATCACCCACTC | |  | IS*Pa21* | 3611 |  |
| MS03-D | CATCCGACGCTTGGACTATT | |  | *urf2* |  | This study |
| HS1157 | CGATCACCGAATTGAAGC | |  | IS*6100* | 4505 |  |
| HS1275 | CGCTGATAGAAACGGATGGT | |  | *merR* Tn*6163* |  |  |
| HS1264 | TGCCTTCCTCGATAACCAAC | |  | *merA* Tn*6163* | 3034 |  |
| MS02-D | CTCATGAGCGAAGTGCAAAA | |  |  |  | This study |
|  | | **ExoU Island A Bridging Primers** | | | | |
| MS05-D | CTGGGCCAGTAGGTAGGC | |  | EXA2 | 625 | This study |
| MS06-D | CCTACGCACTCAAGGTACGG | |  | Between EXA2 and EXA3 |  |  |
| MS07-D | CTACAAGCCGAGGAAGAACG | |  |  | 1666 |  |
| MS08-D | ACACCCGGTAGTCATCAAGC | |  | PACL_0397 |  |  |
| MS09-D | CAGGGAATGGATCGTGTTCT | |  | PACL_0423 | 1837 |  |
| MS10-D | CCACTGCTTAGGGCTAGTCG | |  |  |  |  |
| MS11-D | ATCGGTCACAAACGACTTCC | |  | Outside of GI2 | 2541 |  |
| MS12-D | TGACGAACGACTTCTTGTGC | |  | GI2 – ORF01 |  |  |
| MS13-D | CCAGGGATTCTTGTCTTCCA | |  | Outisde of GI2 | 2877 |  |
| MS14-D | GGCAAGGTGGTACGGATCTA | |  | GI2 – after ORF88 |  |  |

Table S2: Sequence Assembly statistics

| Sample | RNS_PA1 | RNS_PA46 | RNS_PAE05 |
| --- | --- | --- | --- |
| Coverage | 38 | 35 | 35 |
| Number of scaffolds | 152 | 179 | 180 |
| Genome size | 7034586 | 7122869 | 7039584 |
| Max scaffold length | 292513 | 291332 | 284805 |
| Scaffold N50 | 111819 | 99078 | 99775 |
| Scaffold N90 | 35930 | 28003 | 26302 |
| Average Length | 46280.2 | 39792.6 | 39108.8 |
| GC Content | 0.659143 | 0.658502 | 0.659082 |

Table S3 Annotation of GI2

| ORF | Annotation Result | Length (bp) | Start | Stop | pBLAST result (query%/ID%) | Accession |
| --- | --- | --- | --- | --- | --- | --- |
| DR | Direct Repeat | 12 | 1 | 12 |  |  |
| ORF1 | intergrase | 1929 | 216 | 2144 | 100/100 | WP_003158602.1 |
| ORF2 | hypothetical protein | 756 | 2696 | 3451 | 100/100 | WP_003158603.1 |
| ORF3 | Transcriptional regulator | 213 | 3570 | 3782 | 100/100 | WP_003158604.1 |
| ORF4 | cobyrinic acid a,c-diamide synthase | 876 | 3825 | 4700 | 100/100 | WP_003158605.1 |
| ORF5 | hypothetical protein | 249 | 4684 | 4932 | 100/100 | WP_003158606.1 |
| ORF6 | hypothetical protein | 1629 | 4925 | 6553 | 100/100 | WP_003158607.1 |
| ORF7 | coproporphyrinogen III oxidase | 561 | 6569 | 7129 | 100/100 | WP_003158608.1 |
| ORF8 | hypothetical protein | 1245 | 7133 | 8377 | 100/100 | WP_003158609.1 |
| ORF9 | hypothetical protein | 786 | 8695 | 9480 | 100/100 | WP_003158610.1 |
| ORF10 | integrase | 528 | 9477 | 10005 | 100/100 | WP_003158611.1 |
| ORF11 | single-stranded DNA-binding protein | 441 | 10078 | 10518 | 100/100 | WP_003158612.1 |
| ORF12 | DNA topoisomerase III | 2025 | 10788 | 12812 | 100/100 | WP_003158613.1 |
| ORF13 | hypothetical protein | 246 | 13337 | 13582 | 100/100 | WP_003158614.1 |
| ORF14 | hypothetical protein Daci_4152 | 213 | 13911 | 14123 | 100/100 | YP_001565168.1\| |
| ORF15 | hypothetical protein | 393 | 14145 | 14537 | 100/100 | WP_003158618.1 |
| ORF16 | ABC transporter substrate-binding protein | 738 | 14750 | 15487 | 100/100 | WP_003158619.1 |
| ORF17 | uridylate kinase | 279 | 15582 | 15860 | 100/100 | WP_003158620.1 |
| ORF18 | hypothetical protein | 813 | 16140 | 16952 | 100/100 | WP_003158621.1 |
| ORF19 | hypothetical protein | 915 | 17329 | 18243 | 100/100 | WP_003158622.1 |
| ORF20 | hypothetical protein | 699 | 18298 | 18996 | 100/100 | WP_003158623.1 |
| ORF21 | hypothetical protein | 402 | 19091 | 19492 | 100/100 | WP_003158624.1 |
| ORF22 | hypothetical protein | 651 | 19575 | 20225 | 100/100 | WP_003158625.1 |
| ORF23 | O-methyl transferase | 1122 | 20290 | 21411 | 100/100 | WP_003158626.1 |
| ORF24 | hypothetical protein | 312 | 21512 | 21823 | 100/100 | WP_003158627.1 |
| ORF25 | DEAD/DEAH box helicase | 2860 | 21961 | 24240 | 100/100 | WP_003158628.1 |
| ORF26 | hypothetical protein | 579 | 24474 | 25052 | 100/100 | WP_003158629.1 |
| ORF27 | hypothetical protein | 642 | 25049 | 25690 | 100/100 | WP_003158630.1 |
| ORF28 | hypothetical protein | 738 | 25705 | 26442 | 100/100 | WP_003158631.1 |
| ORF29 | lytic transglycosylase | 606 | 26424 | 27029 | 100/100 | WP_003158632.1 |
| ORF30 | hypothetical protein | 549 | 27026 | 27574 | 100/100 | WP_003158633.1 |
| ORF31 | conjugal transfer protein TraG | 2061 | 27705 | 29765 | 100/100 | WP_003159558.1 |
| ORF32 | membrane protein | 750 | 29762 | 30511 | 100/100 | WP_003158635.1 |
| ORF33 | hypothetical protein | 1371 | 31882 | 30512 | 100/100 | WP_003158636.1 |
| ORF34 | signal peptide protein | 381 | 32044 | 32424 | 100/100 | WP_003158637.1 |
| ORF35 | hypothetical protein Bxe_A1191 | 234 | 32421 | 32654 | 100/100 | YP_559814.1 |
| ORF36 | hypothetical protein | 360 | 32671 | 33030 | 100/100 | WP_003158638.1 |
| ORF37 | hypothetical protein HEAR2015 | 411 | 33043 | 33453 | 100/100 | YP_001100282.1 |
| ORF38 | hypothetical protein | 690 | 33450 | 34139 | 100/100 | WP_003158640.1 |
| ORF39 | hypothetical protein | 933 | 34136 | 35068 | 100/100 | WP_003158641.1 |
| ORF40 | hypothetical protein | 1419 | 35058 | 36476 | 100/100 | WP_003158643.1 |
| ORF41 | conjugal transfer protein | 2871 | 36897 | 39767 | 100/100 | WP_003158645.1 |
| ORF42 | hypothetical protein | 726 | 39810 | 40535 | 100/100 | WP_003158646.1 |
| ORF43 | DNA repair protein RadC | 495 | 40722 | 41216 | 100/100 | WP_003158647.1 |
| ORF44 | hypothetical protein | 447 | 41360 | 41806 | 100/100 | WP_003158648.1 |
| ORF45 | hypothetical protein | 948 | 41803 | 42750 | 100/100 | WP_003158649.1 |
| ORF46 | integrating conjugative element protein | 1395 | 42761 | 44155 | 100/100 | WP_003158650.1 |
| ORF47 | hypothetical protein | 360 | 44152 | 44511 | 100/100 | WP_003158651.1 |
| ORF48 | membrane protein | 1524 | 44527 | 46050 | 100/100 | WP_003158652.1 |
| ORF49 | hypothetical protein | 363 | 46419 | 46057 | 100/100 | WP_003158653.1 |
| ORF50 | hypothetical protein | 462 | 46908 | 46447 | 100/100 | WP_003158654.1 |
| ORF51 | AbrB family transcriptional regulator | 318 | 47222 | 46905 | 100/100 | WP_003158655.1 |
| ORF52 | hypothetical protein | 1863 | 47534 | 49396 | 100/100 | WP_003158656.1 |
| ORF53 | hypothetical protein | 582 | 49988 | 49407 | 100/100 | WP_003158657.1 |
|  | **Tn6163 insertion** |  | 50136 | 50140 |  |  |
| ORF54 | ATP-dependent OLD family endonuclease | 1881 | 52699 | 50819 | 100/100 | WP_003158915.1 |
| ORF55 | type III restriction endonuclease subunit R | 3006 | 55761 | 52756 | 100/100 | WP_003160810.1 |
| ORF56 | adenine methyltransferase | 2157 | 57930 | 55774 | 100/100 | WP_003158913.1 |
| ORF57 | hypothetical protein | 807 | 60869 | 60063 | 100/100 | WP_003158911.1 |
| ORF58 | helicase | 3222 | 64087 | 60686 | 100/100 | WP_003160812.1 |
| ORF59 | hypothetical protein | 909 | 65008 | 64100 | 100/100 | WP_003158909.1 |
| ORF60 | D-alanyl-D-alanine endopeptidase | 891 | 65288 | 66178 | 100/100 | WP_003158908.1 |
| ORF61 | DNA-binding protein | 318 | 66313 | 66630 | 100/100 | WP_023464296.1 |
| ORF62 | ATPase | 1032 | 66623 | 67654 | 100/100 | WP_003158906.1 |
| ORF63 | hypothetical protein | 2511 | 67632 | 70142 | 100/100 | WP_003160813.1 |
| ORF64 | XRE family transcriptional regulator | 147 | 70387 | 70241 | 100/100 | WP_003160814.1 |
| ORF65 | hypothetical protein NCGM2_1380 | 477 | 72087 | 71611 | 100/100 | YP_005979631.1 |
| ORF66 | hypothetical protein NCGM2_1379 | 294 | 72612 | 72319 | 100/100 | YP_005979630.1 |
| ORF67 | hypothetical protein PA0712 | 258 | 72952 | 73209 | 100/100 | NP_249403.1 |
| ORF68 | hypothetical protein NCGM2_1377 | 507 | 73269 | 73775 | 100/100 | YP_005979628.1 |
| ORF69 | lactoylglutathione lyase | 396 | 74214 | 73819 | 100/100 | YP_005979627.1 |
| ORF70 | hypothetical protein PA0709 | 297 | 74555 | 74259 | 100/100 | NP_249400.1 |
| ORF71 | exotoxin A regulatory protein | 780 | 75811 | 76590 | 100/100 | YP_008887013.1 |
| ORF72 | regB | 228 | 76597 | 76824 | 100/100 | CAA36701.1 |
| ORF73 | chloramphenicol acetyltransferase | 639 | 76683 | 77321 | 100/100 | YP_005979623.1 |
| ORF74 | glycosyl transferase family protein | 903 | 77576 | 78478 | 100/100 | YP_005979621.1 |
| ORF75 | acetyltransferase | 621 | 78512 | 79132 | 100/100 | WP_003085401.1 |
| ORF76 | amidase | 1395 | 79259 | 80653 | 100/100 | YP_005979620.1 |
| ORF77 | MFS family transporter | 1317 | 80724 | 82040 | 100/100 | YP_005979619.1 |
| ORF78 | hypothetical protein PA0702 | 906 | 82119 | 83024 | 100/100 | NP_249393.1 |
| ORF79 | hypothetical protein PA14_55240 | 666 | 83672 | 83007 | 100/100 | YP_792593.1 |
| ORF80 | transcriptional regulator | 987 | 84730 | 83744 | 100/100 | YP_005979616.1 |
| ORF81 | hypothetical protein NCGM2_1364 | 426 | 85165 | 84740 | 100/100 | YP_005979615.1 |
| ORF82 | putative peptidyl-prolyl cis-trans isomerase, PpiC-type | 945 | 86109 | 85165 | 100/100 | YP_005976922.1 |
| ORF83 | hypothetical protein PA0698 | 480 | 86593 | 86114 | 100/100 | NP_249389.1 |
| ORF84 | hypothetical protein PA0697 | 663 | 87284 | 86622 | 100/100 | NP_249388.1 |
| ORF85 | hypothetical protein PA14_55320 | 1707 | 89025 | 87319 | 100/100 | YP_792599.1 |
| ORF86 | hypothetical protein NCGM2_1359 | 747 | 89839 | 89093 | 100/100 | YP_005979610.1 |
| ORF87 | transporter ExbD | 420 | 90267 | 89848 | 100/100 | NP_249385.1 |
| ORF88 | transport protein ExbB2 | 1848 | 92125 | 90278 | 100/100 | YP_792602.1 |
| DR | Direct Repeat | 12 | 94357 | 94368 |  |  |

Table S4: RAST Annotation of the regions that are exclusive to genomes RNS_PA1, RNS_PA46 and RNS_PAE05

| contig_id | feature_id | function |
| --- | --- | --- |
| RNS_PA1 |  |  |
| scaffold109.1\|size2035 | [fig\|287.659.peg.1012](http://rast.nmpdr.org/seedviewer.cgi?page=Annotation&feature=fig\|287.659.peg.1012) | FIG00954937: hypothetical protein |
| scaffold109.1\|size2035 | [fig\|287.659.peg.1013](http://rast.nmpdr.org/seedviewer.cgi?page=Annotation&feature=fig\|287.659.peg.1013) | hypothetical protein |
| scaffold109.1\|size2035 | [fig\|287.659.peg.1014](http://rast.nmpdr.org/seedviewer.cgi?page=Annotation&feature=fig\|287.659.peg.1014) | hypothetical protein |
| scaffold109.1\|size2035 | [fig\|287.659.peg.1015](http://rast.nmpdr.org/seedviewer.cgi?page=Annotation&feature=fig\|287.659.peg.1015) | hypothetical protein |
| scaffold109.1\|size2035 | [fig\|287.659.peg.1016](http://rast.nmpdr.org/seedviewer.cgi?page=Annotation&feature=fig\|287.659.peg.1016) | phage holin, lambda family |
| scaffold112.1\|size1744 | [fig\|287.659.peg.1169](http://rast.nmpdr.org/seedviewer.cgi?page=Annotation&feature=fig\|287.659.peg.1169) | Phage tail fiber protein |
| scaffold115.1\|size1674 | [fig\|287.659.peg.1171](http://rast.nmpdr.org/seedviewer.cgi?page=Annotation&feature=fig\|287.659.peg.1171) | Mobile element protein |
| scaffold118.1\|size1603 | [fig\|287.659.peg.1174](http://rast.nmpdr.org/seedviewer.cgi?page=Annotation&feature=fig\|287.659.peg.1174) | Dihydropteroate synthase (EC 2.5.1.15) |
| scaffold123.1\|size1236 | [fig\|287.659.peg.1320](http://rast.nmpdr.org/seedviewer.cgi?page=Annotation&feature=fig\|287.659.peg.1320) | Mobile element protein |
| scaffold123.1\|size1236 | [fig\|287.659.peg.1321](http://rast.nmpdr.org/seedviewer.cgi?page=Annotation&feature=fig\|287.659.peg.1321) | Mobile element protein |
| scaffold132.1\|size817 | [fig\|287.659.peg.1454](http://rast.nmpdr.org/seedviewer.cgi?page=Annotation&feature=fig\|287.659.peg.1454) | hypothetical protein |
| scaffold136.1\|size727 | [fig\|287.659.peg.1455](http://rast.nmpdr.org/seedviewer.cgi?page=Annotation&feature=fig\|287.659.peg.1455) | hypothetical protein |
| scaffold143.1\|size611 | [fig\|287.659.peg.1556](http://rast.nmpdr.org/seedviewer.cgi?page=Annotation&feature=fig\|287.659.peg.1556) | Phage protein |
| scaffold149.1\|size548 | [fig\|287.659.peg.1558](http://rast.nmpdr.org/seedviewer.cgi?page=Annotation&feature=fig\|287.659.peg.1558) | Mobile element protein |
| RNS_PA46 | |  |
| scaffold79.1\|size24310 | [fig\|287.660.peg.6186](http://rast.nmpdr.org/seedviewer.cgi?page=Annotation&feature=fig\|287.660.peg.6186) | Type IV secretory pathway, VirD4 components |
| scaffold79.1\|size24310 | [fig\|287.660.peg.6187](http://rast.nmpdr.org/seedviewer.cgi?page=Annotation&feature=fig\|287.660.peg.6187) | putative membrane protein |
| scaffold79.1\|size24310 | [fig\|287.660.peg.6188](http://rast.nmpdr.org/seedviewer.cgi?page=Annotation&feature=fig\|287.660.peg.6188) | putative DNA helicase |
| scaffold79.1\|size24310 | [fig\|287.660.peg.6189](http://rast.nmpdr.org/seedviewer.cgi?page=Annotation&feature=fig\|287.660.peg.6189) | Transcriptional regulator, TetR family |
| scaffold79.1\|size24310 | [fig\|287.660.peg.6190](http://rast.nmpdr.org/seedviewer.cgi?page=Annotation&feature=fig\|287.660.peg.6190) | Lysophospholipase (EC 3.1.1.5) |
| scaffold79.1\|size24310 | [fig\|287.660.peg.6191](http://rast.nmpdr.org/seedviewer.cgi?page=Annotation&feature=fig\|287.660.peg.6191) | Aconitase B |
| scaffold79.1\|size24310 | [fig\|287.660.peg.6192](http://rast.nmpdr.org/seedviewer.cgi?page=Annotation&feature=fig\|287.660.peg.6192) | Candidate type III effector Hop protein |
| scaffold79.1\|size24310 | [fig\|287.660.peg.6193](http://rast.nmpdr.org/seedviewer.cgi?page=Annotation&feature=fig\|287.660.peg.6193) | hypothetical protein |
| scaffold79.1\|size24310 | [fig\|287.660.peg.6194](http://rast.nmpdr.org/seedviewer.cgi?page=Annotation&feature=fig\|287.660.peg.6194) | FIG00955871: hypothetical protein |
| scaffold79.1\|size24310 | [fig\|287.660.peg.6195](http://rast.nmpdr.org/seedviewer.cgi?page=Annotation&feature=fig\|287.660.peg.6195) | FIG00953508: hypothetical protein |
| scaffold79.1\|size24310 | [fig\|287.660.peg.6196](http://rast.nmpdr.org/seedviewer.cgi?page=Annotation&feature=fig\|287.660.peg.6196) | Possible exported protein |
| scaffold79.1\|size24310 | [fig\|287.660.peg.6197](http://rast.nmpdr.org/seedviewer.cgi?page=Annotation&feature=fig\|287.660.peg.6197) | FIG00958851: hypothetical protein |
| scaffold79.1\|size24310 | [fig\|287.660.peg.6198](http://rast.nmpdr.org/seedviewer.cgi?page=Annotation&feature=fig\|287.660.peg.6198) | putative exported protein |
| scaffold79.1\|size24310 | [fig\|287.660.peg.6199](http://rast.nmpdr.org/seedviewer.cgi?page=Annotation&feature=fig\|287.660.peg.6199) | putative lipoprotein |
| scaffold79.1\|size24310 | [fig\|287.660.peg.6200](http://rast.nmpdr.org/seedviewer.cgi?page=Annotation&feature=fig\|287.660.peg.6200) | Type IV secretory pathway, VirB4 components |
| scaffold79.1\|size24310 | [fig\|287.660.peg.6201](http://rast.nmpdr.org/seedviewer.cgi?page=Annotation&feature=fig\|287.660.peg.6201) | FIG00960652: hypothetical protein |
| scaffold79.1\|size24310 | [fig\|287.660.peg.6202](http://rast.nmpdr.org/seedviewer.cgi?page=Annotation&feature=fig\|287.660.peg.6202) | Protein-disulfide isomerase |
| scaffold79.1\|size24310 | [fig\|287.660.peg.6203](http://rast.nmpdr.org/seedviewer.cgi?page=Annotation&feature=fig\|287.660.peg.6203) | Protein-disulfide isomerase |
| scaffold79.1\|size24310 | [fig\|287.660.peg.6204](http://rast.nmpdr.org/seedviewer.cgi?page=Annotation&feature=fig\|287.660.peg.6204) | FIG00962909: hypothetical protein |
| scaffold79.1\|size24310 | [fig\|287.660.peg.6205](http://rast.nmpdr.org/seedviewer.cgi?page=Annotation&feature=fig\|287.660.peg.6205) | FIG00956406: hypothetical protein |
| scaffold79.1\|size24310 | [fig\|287.660.peg.6206](http://rast.nmpdr.org/seedviewer.cgi?page=Annotation&feature=fig\|287.660.peg.6206) | FIG00959181: hypothetical protein |
| scaffold79.1\|size24310 | [fig\|287.660.peg.6207](http://rast.nmpdr.org/seedviewer.cgi?page=Annotation&feature=fig\|287.660.peg.6207) | FIG00953975: hypothetical protein |
| scaffold79.1\|size24310 | [fig\|287.660.peg.6208](http://rast.nmpdr.org/seedviewer.cgi?page=Annotation&feature=fig\|287.660.peg.6208) | putative membrane protein |
| scaffold79.1\|size24310 | [fig\|287.660.peg.6209](http://rast.nmpdr.org/seedviewer.cgi?page=Annotation&feature=fig\|287.660.peg.6209) | Predicted transcriptional regulators containing the CopG/Arc/MetJ DNA-binding domain |
| scaffold79.1\|size24310 | [fig\|287.660.peg.6210](http://rast.nmpdr.org/seedviewer.cgi?page=Annotation&feature=fig\|287.660.peg.6210) | putative plasmid stablization protein |
| scaffold115.1\|size5352 | [fig\|287.660.peg.1344](http://rast.nmpdr.org/seedviewer.cgi?page=Annotation&feature=fig\|287.660.peg.1344) | hypothetical protein |
| scaffold115.1\|size5352 | [fig\|287.660.peg.1345](http://rast.nmpdr.org/seedviewer.cgi?page=Annotation&feature=fig\|287.660.peg.1345) | Basic proline-rich protein precursor |
| scaffold129.1\|size2662 | [fig\|287.660.peg.1381](http://rast.nmpdr.org/seedviewer.cgi?page=Annotation&feature=fig\|287.660.peg.1381) | hypothetical protein |
| scaffold129.1\|size2662 | [fig\|287.660.peg.1382](http://rast.nmpdr.org/seedviewer.cgi?page=Annotation&feature=fig\|287.660.peg.1382) | Secreted protein Hcp |
| scaffold136.1\|size1784 | [fig\|287.660.peg.1523](http://rast.nmpdr.org/seedviewer.cgi?page=Annotation&feature=fig\|287.660.peg.1523) | Secreted protein Hcp |
| scaffold147.1\|size1236 | [fig\|287.660.peg.1653](http://rast.nmpdr.org/seedviewer.cgi?page=Annotation&feature=fig\|287.660.peg.1653) | Mobile element protein |
| scaffold147.1\|size1236 | [fig\|287.660.peg.1654](http://rast.nmpdr.org/seedviewer.cgi?page=Annotation&feature=fig\|287.660.peg.1654) | Mobile element protein |
| scaffold154.1\|size817 | [fig\|287.660.peg.1772](http://rast.nmpdr.org/seedviewer.cgi?page=Annotation&feature=fig\|287.660.peg.1772) | hypothetical protein |
| scaffold158.1\|size732 | [fig\|287.660.peg.1773](http://rast.nmpdr.org/seedviewer.cgi?page=Annotation&feature=fig\|287.660.peg.1773) | Phenazine biosynthesis protein PhzA |
| scaffold166.1\|size614 | [fig\|287.660.peg.1893](http://rast.nmpdr.org/seedviewer.cgi?page=Annotation&feature=fig\|287.660.peg.1893) | hypothetical protein |
| scaffold167.1\|size611 | [fig\|287.660.peg.1894](http://rast.nmpdr.org/seedviewer.cgi?page=Annotation&feature=fig\|287.660.peg.1894) | hypothetical protein |
| scaffold170.1\|size583 | [fig\|287.660.peg.2024](http://rast.nmpdr.org/seedviewer.cgi?page=Annotation&feature=fig\|287.660.peg.2024) | FIG00953426: hypothetical protein |
| scaffold171.1\|size581 | [fig\|287.660.peg.2025](http://rast.nmpdr.org/seedviewer.cgi?page=Annotation&feature=fig\|287.660.peg.2025) | Phenazine biosynthesis protein PhzB |
| scaffold173.1\|size548 | [fig\|287.660.peg.2026](http://rast.nmpdr.org/seedviewer.cgi?page=Annotation&feature=fig\|287.660.peg.2026) | Mobile element protein |
| scaffold174.1\|size547 | [fig\|287.660.peg.2027](http://rast.nmpdr.org/seedviewer.cgi?page=Annotation&feature=fig\|287.660.peg.2027) | FIG00953426: hypothetical protein |
| scaffold175.1\|size537 | [fig\|287.660.peg.2028](http://rast.nmpdr.org/seedviewer.cgi?page=Annotation&feature=fig\|287.660.peg.2028) | hypothetical protein |
| scaffold179.1\|size502 | [fig\|287.660.peg.2029](http://rast.nmpdr.org/seedviewer.cgi?page=Annotation&feature=fig\|287.660.peg.2029) | Transcriptional regulator, ArsR family |
| RNS_PAE05 | |  |
| scaffold125.1\|size3994 | [fig\|287.661.peg.1397](http://rast.nmpdr.org/seedviewer.cgi?page=Annotation&feature=fig\|287.661.peg.1397) | 2-keto-3-deoxy-D-arabino-heptulosonate-7-phosphate synthase II (EC 2.5.1.54) # AroA II |
| scaffold125.1\|size3994 | [fig\|287.661.peg.1398](http://rast.nmpdr.org/seedviewer.cgi?page=Annotation&feature=fig\|287.661.peg.1398) | Isochorismatase (EC 3.3.2.1) of siderophore biosynthesis |
| scaffold125.1\|size3994 | [fig\|287.661.peg.1399](http://rast.nmpdr.org/seedviewer.cgi?page=Annotation&feature=fig\|287.661.peg.1399) | 2-Amino-2-deoxy-isochorismate synthase (EC 4.1.3.-) # TrpAa/TrpAb-PhzE type |
| scaffold137.1\|size2035 | [fig\|287.661.peg.1547](http://rast.nmpdr.org/seedviewer.cgi?page=Annotation&feature=fig\|287.661.peg.1547) | FIG00954937: hypothetical protein |
| scaffold137.1\|size2035 | [fig\|287.661.peg.1548](http://rast.nmpdr.org/seedviewer.cgi?page=Annotation&feature=fig\|287.661.peg.1548) | hypothetical protein |
| scaffold137.1\|size2035 | [fig\|287.661.peg.1549](http://rast.nmpdr.org/seedviewer.cgi?page=Annotation&feature=fig\|287.661.peg.1549) | hypothetical protein |
| scaffold137.1\|size2035 | [fig\|287.661.peg.1550](http://rast.nmpdr.org/seedviewer.cgi?page=Annotation&feature=fig\|287.661.peg.1550) | hypothetical protein |
| scaffold137.1\|size2035 | [fig\|287.661.peg.1551](http://rast.nmpdr.org/seedviewer.cgi?page=Annotation&feature=fig\|287.661.peg.1551) | phage holin, lambda family |
| scaffold142.1\|size1708 | [fig\|287.661.peg.1643](http://rast.nmpdr.org/seedviewer.cgi?page=Annotation&feature=fig\|287.661.peg.1643) | Basic proline-rich protein precursor |
| scaffold144.1\|size1649 | [fig\|287.661.peg.1644](http://rast.nmpdr.org/seedviewer.cgi?page=Annotation&feature=fig\|287.661.peg.1644) | Secreted protein Hcp |
| scaffold148.1\|size1236 | [fig\|287.661.peg.1646](http://rast.nmpdr.org/seedviewer.cgi?page=Annotation&feature=fig\|287.661.peg.1646) | Mobile element protein |
| scaffold148.1\|size1236 | [fig\|287.661.peg.1647](http://rast.nmpdr.org/seedviewer.cgi?page=Annotation&feature=fig\|287.661.peg.1647) | Mobile element protein |
| scaffold150.1\|size1064 | [fig\|287.661.peg.1768](http://rast.nmpdr.org/seedviewer.cgi?page=Annotation&feature=fig\|287.661.peg.1768) | Phenazine biosynthesis protein PhzA |
| scaffold150.1\|size1064 | [fig\|287.661.peg.1769](http://rast.nmpdr.org/seedviewer.cgi?page=Annotation&feature=fig\|287.661.peg.1769) | Phenazine biosynthesis protein PhzB |
| scaffold153.1\|size906 | [fig\|287.661.peg.1770](http://rast.nmpdr.org/seedviewer.cgi?page=Annotation&feature=fig\|287.661.peg.1770) | Mobile element protein |

Table S5. CARD analysis

|  | RNS_PA1 | RNS_PA46 | RNS_PAE05 | NCGM2 | NCGM 1900 |
| --- | --- | --- | --- | --- | --- |
| *sul1*  (sulfonamide) | Present  CARD BlastP 100%  pBLAST 100% | Present  CARD BlastP 100%  pBLAST 100% | Present  CARD BlastP 100%  pBLAST 100% | Present  CARD BlastP 100%  pBLAST 100% | Present  CARD BlastP 100%  pBLAST 99% |
| *sul1*  (sulfonamide) | Present  CARD BlastP 100%  pBLAST 100% | Present  CARD BlastP 100%  pBLAST 100% | Present  CARD BlastP 100%  pBLAST 100% |  | Present  CARD BlastP 100%  pBLAST 99% |
| *sul1*  (sulfonamide) | Present  CARD BlastP 100%  pBLAST 100% | Present  CARD BlastP 100%  pBLAST 100% | Present  CARD BlastP 100%  pBLAST 100% |  | Present  CARD BlastP 100%  pBLAST 100% |
| *bla_OXA-50_*  (beta-lactamase) | Present  CARD BlastP 99.24%  pBLAST 100% | Present  CARD BLASTP 99.24%  pBLAST 100% | Present  CARD BLASTP 99.24%  pBLAST 100% | Present  CARD BLASTP 99.24%  pBLAST 100% | Present  CARD BLASTP 99.24%  pBLAST 100% |
| *bla_PDC-2_*  (beta-lactamase) | Present  CARD BlastP 99.75%  pBLAST 100% | Present  CARD BlastP 99.75%  pBLAST 100% | Present  CARD BlastP 99.75%  pBLAST 100% | Present  CARD BlastP 99.75%  pBLAST 100% | Present  CARD BlastP 99.75%  pBLAST 100% |
| *aph(3’)-IIb*  (aminoglycoside) | Present  CARD BlastP 98.88%  pBLAST 100% | Present  CARD BlastP 98.88%  pBLAST 100% | Present  CARD BlastP 98.88%  pBLAST 100% | Present  CARD BlastP 98.88%  pBLAST 100% | Present  CARD BlastP 98.88%  pBLAST 100% |
| aac(6')Ib  (aminoglycoside) | Present  CARD BlastP 100%  pBLAST 100% |  |  |  |  |
| *aadA*  (aminoglycoside) |  |  |  | Present  CARD BlastP 100%  pBLAST 100% |  |
| *catB7*  (chloramphenicol) | Present  CARD BlastP 98.58%  pBLAST 100% | Present  CARD BlastP 98.58%  pBLAST 100% | Present  CARD BlastP 98.58%  pBLAST 100% | Present  CARD BlastP 98.58%  pBLAST 100% | Present  CARD BlastP 98.58%  pBLAST 100% |
| *mdsB*  (efflux pump) | Present  CARD BlastP 81.29%  pBLAST 100% | Present  CARD BlastP 81.29%  pBLAST 100% |  |  | Present  CARD BlastP 69.46%  pBLAST 100% |
| *tetG*  (efflux pump) |  | Present nonfunctional  CARD BlastP 94.37%  pBLAST 99% |  |  |  |
| *mexD*  (efflux pump) | Present  CARD BlastP 99.81%  pBLAST 100% | Present  CARD BlastP 99.81%  pBLAST 100% | Present  CARD BlastP 99.81%  pBLAST 100% | Present  CARD BlastP 99.81%  pBLAST 100% | Present  CARD BlastP 99.81%  pBLAST 100% |
| *mexB*  (efflux pump) | Present  CARD BlastP 99.62%  pBLAST 100% | Present  CARD BlastP 99.62%  pBLAST 100% | Present  CARD BlastP 99.62%  pBLAST 100% | Present  CARD BlastP 99.62%  pBLAST 100% | Present  CARD BlastP 99.62%  pBLAST 100% |
| *mexF*  (efflux pump) | Present  CARD BlastP 100%  pBLAST 100% | Present  CARD BlastP 100%  pBLAST 100% | Present  CARD BlastP 100%  pBLAST 100% | Present  CARD BlastP 100%  pBLAST 100% | Present  CARD BlastP 100%  pBLAST 100% |
| *amrB*  (efflux pump) | Present  CARD BlastP 99.90%  pBLAST 100% | Present  CARD BlastP 99.90%  pBLAST 100% | Present  CARD BlastP 99.90%  pBLAST 100% | Present  CARD BlastP 99.90%  pBLAST 100% | Present  CARD BlastP 99.90%  pBLAST 100% |
| *mexA*  (efflux pump) | Present  CARD BlastP 100%  pBLAST 100% | Present  CARD BlastP 100%  pBLAST 100% | Present  CARD BlastP 100%  pBLAST 100% | Present  CARD BlastP 100%  pBLAST 100% | Present  CARD BlastP 100%  pBLAST 100% |
| *recG**  (Requirement for mexA function) | Present  pBLAST 100% | Present  pBLAST100% | Present  pBLAST 100% | Present  pBLAST 100% | Present  pBLAST 100% |
| *mexI*  (efflux pump) | Present  CARD BlastP 99.90%  pBLAST 100% | Present  CARD BlastP 99.90%  pBLAST 100% | Present  CARD BlastP 99.90%  pBLAST 100% | Present  CARD BlastP 99.90%  pBLAST 100% | Present  CARD BlastP 99.90%  pBLAST 100% |
| *oprM*  (efflux pump) | Present  CARD BlastP 100%  pBLAST 100% | Present  CARD BlastP 100%  pBLAST 100% | Present  CARD BlastP 100%  pBLAST 100% | Present  CARD BlastP 100%  pBLAST 100% | Present  CARD BlastP 100%  pBLAST 100% |
| *mexC*  (efflux pump) | Present  CARD BlastP 98.87%  pBLAST 100% | Present  CARD BlastP 98.87%  pBLAST 100% | Present  CARD BlastP 98.87%  pBLAST 100% | Present  CARD BlastP 98.87%  pBLAST 100% | Present  CARD BlastP 98.66%  pBLAST 99% |
| *mexR*  (efflux pump) |  | Present  CARD BlastP 99.32%  pBLAST 100% | Present  CARD BlastP 99.32%  pBLAST 100% | Present  CARD BlastP 99.32%  pBLAST 100% | Present  CARD BlastP 99.64%  pBLAST 100% |
| *oprJ*  (efflux pump) | Present  CARD BlastP  99.16%  pBLAST 100% | Present  CARD BlastP 99.16%  pBLAST 100% | Present  CARD BlastP 99.16%  pBLAST 100% | Present  CARD BlastP 99.16%  pBLAST 100% | Present  CARD BlastP 99.16%  pBLAST 100% |
| *mexE*  (efflux pump) | Present  CARD BlastP 99.28%  pBLAST 100% | Present  CARD BlastP 99.28%  pBLAST 100% | Present  CARD BlastP 99.28%  pBLAST 100% | Present  CARD BlastP 99.28%  pBLAST 100% | Present  CARD BlastP 99.28%  pBLAST 100% |
| *oprN*  (efflux pump) | Present  CARD BlastP 99.79%  pBLAST 100% | Present  CARD BlastP 99.79%  pBLAST 100% | Present  CARD BlastP 99.79%  pBLAST 100% | Present  CARD BlastP 99.79%  pBLAST 100% | Present  CARD BlastP 99.79%  pBLAST 100% |
| *mexG*  (efflux pump) | Present  CARD BlastP 100%  pBLAST 100% | Present  CARD BlastP 100%  pBLAST 100% | Present  CARD BlastP 100%  pBLAST 100% | Present  CARD BlastP 100%  pBLAST 100% | Present  CARD BlastP 100%  pBLAST 100% |
| *mexH*  (efflux pump) | Present  CARD BlastP 99.73%  pBLAST 100% | Present  CARD BlastP 99.73%  pBLAST 100% | Present  CARD BlastP 99.73%  pBLAST 100% | Present  CARD BlastP 99.73%  pBLAST 100% | Present  CARD BlastP 99.73%  pBLAST 100% |
| *opmD*  (efflux pump) | Present  CARD BlastP 99.38%  pBLAST 100% | Present  CARD BlastP 99.38%  pBLAST 100% | Present  CARD BlastP 99.38%  pBLAST 100% | Present  CARD BlastP 99.38%  pBLAST 100% | Present  CARD BlastP 99.38%  pBLAST 100% |
| *mexS*  (efflux pump) | Present  CARD BlastP 100%  pBLAST 100% | Present  CARD BlastP 100%  pBLAST 100% | Present  CARD BlastP 100%  pBLAST 100% | Present  CARD BlastP 100%  pBLAST 100% | Present  CARD BlastP 100%  pBLAST 100% |
| *nalC*  (efflux pump) | Present  CARD BlastP 98.59%  pBLAST 100% | Present  CARD BlastP 98.59%  pBLAST 100% | Present  CARD BlastP 98.59%  pBLAST 100% | Present  CARD BlastP 98.59%  pBLAST 100% | Present  CARD BlastP 98.59%  pBLAST 100% |
| *nalD*  (efflux pump) | Present  CARD BlastP 100%  pBLAST 100% | Present  CARD BlastP 100%  pBLAST 100% | Present  CARD BlastP 100%  pBLAST 100% | Present  CARD BlastP 100%  pBLAST 100% | Present  CARD BlastP 100%  pBLAST 100% |
| *nfxB*  (efflux pump) | Present  CARD BlastP 100%  pBLAST 100% | Present  CARD BlastP 100%  pBLAST 100% | Present  CARD BlastP 100%  pBLAST 100% | Present  CARD BlastP 100%  pBLAST 100% | Present  CARD BlastP 100%  pBLAST 100% |
| *amrA*  (efflux pump) | Present  CARD BlastP 98.99%  pBLAST 100% | Present  CARD BlastP 98.99%  pBLAST 100% | Present  CARD BlastP 98.99%  pBLAST 100% |  | Present  CARD BlastP 98.99%  pBLAST 100% |
| *floR*  (efflux pump) |  | Present  CARD pBLAST 92.82%  pBLAST 100% | Present  CARD pBLAST 92.82%  pBLAST 100% |  |  |

**recG* was not covered by CARD but is necessary for mexA function (ref)
