# Supplementary material for: Genomic islands 1 and 2 play key roles in the evolution of extensively drug-resistant ST235 isolates of Pseudomonas aeruginosa
Source: Open Biol. 2016 Mar 9;6(3):150175. doi: 10.1098/rsob.150175 (PMC4821235; doi:10.1098/rsob.150175)
Supplement: Supplementary Figures [file rsob150175supp2.pdf]

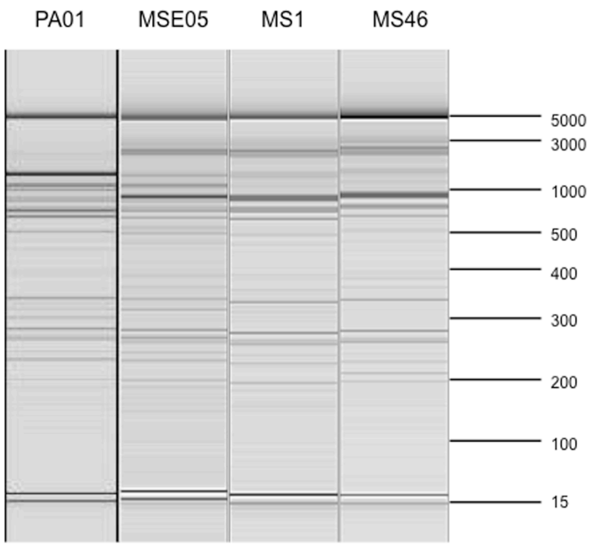


Figure S1: RAPD profiles of the three *P. aeruginosa* isolates

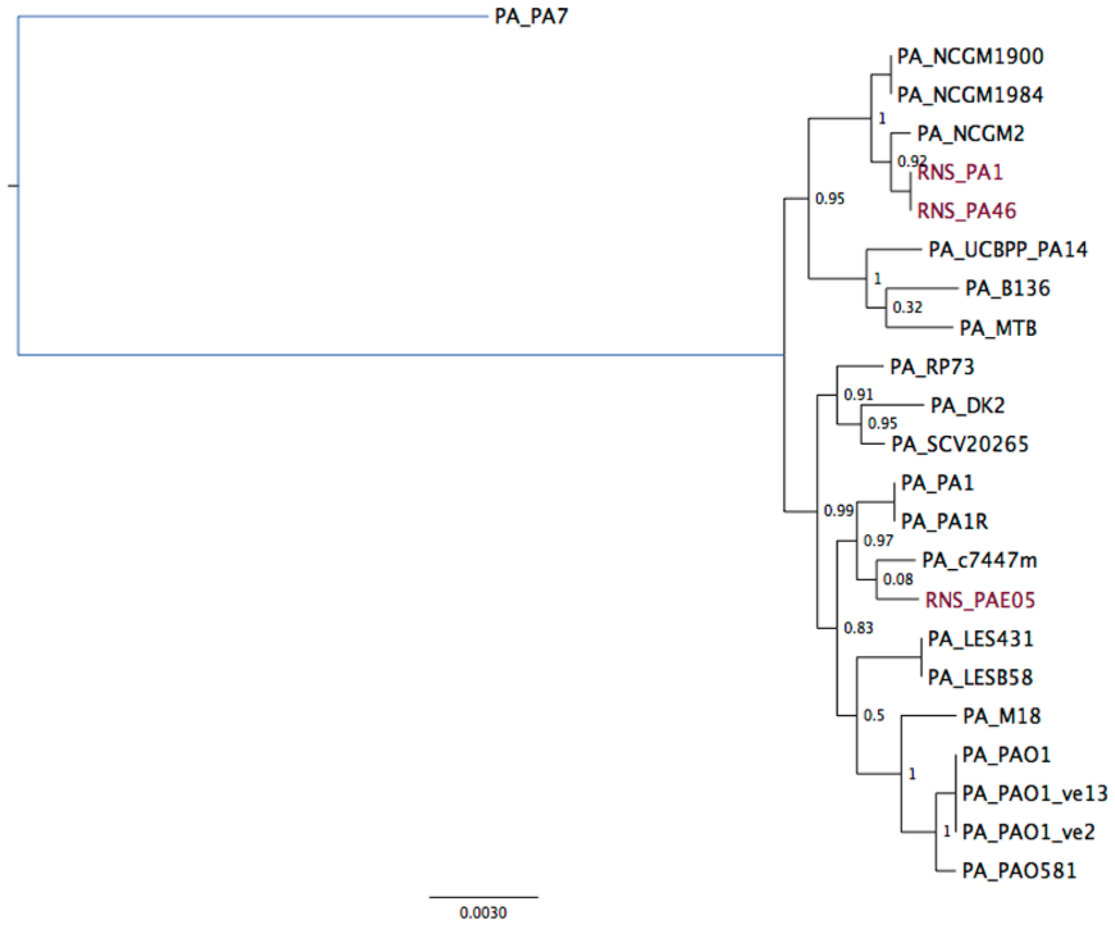


Figure S2: Phylogenetic tree generated using PhyloSift.

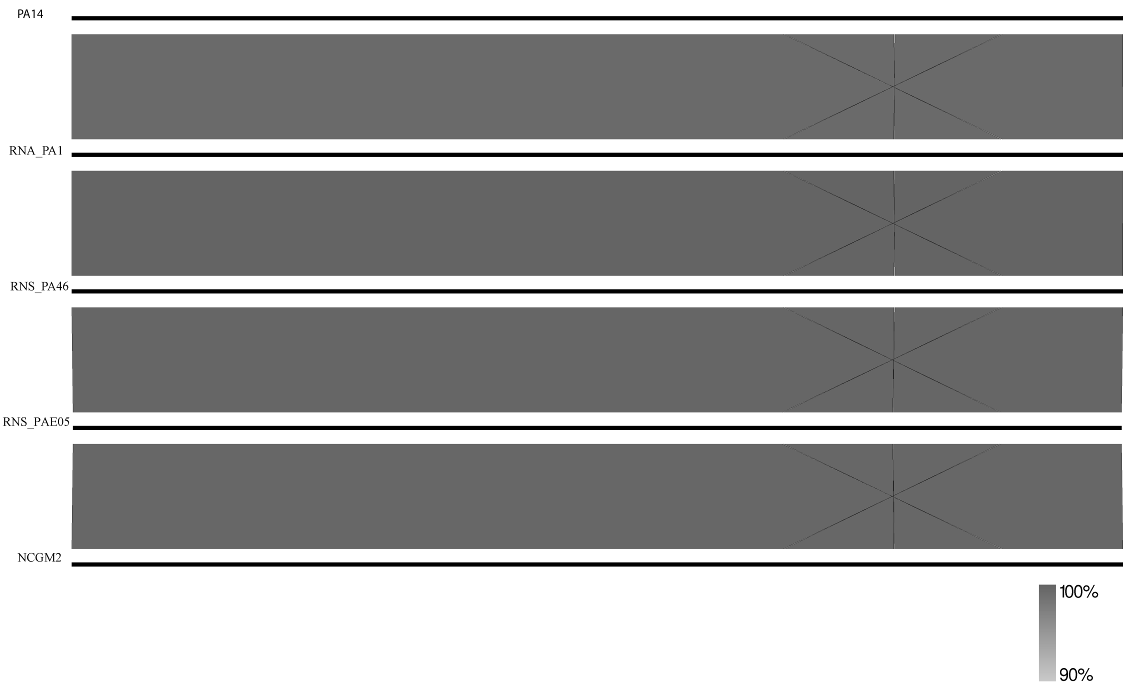


Figure S3: Alignment of the genomic segments encoding the structural genes that form the type III secretion (T3SS) apparatus in *Pseudomonas aeruginosa*. The colour key indicates the percentage of DNA sequence identity across the genomic segment in the three genomes and NCGM2 in comparison to that of PA14.

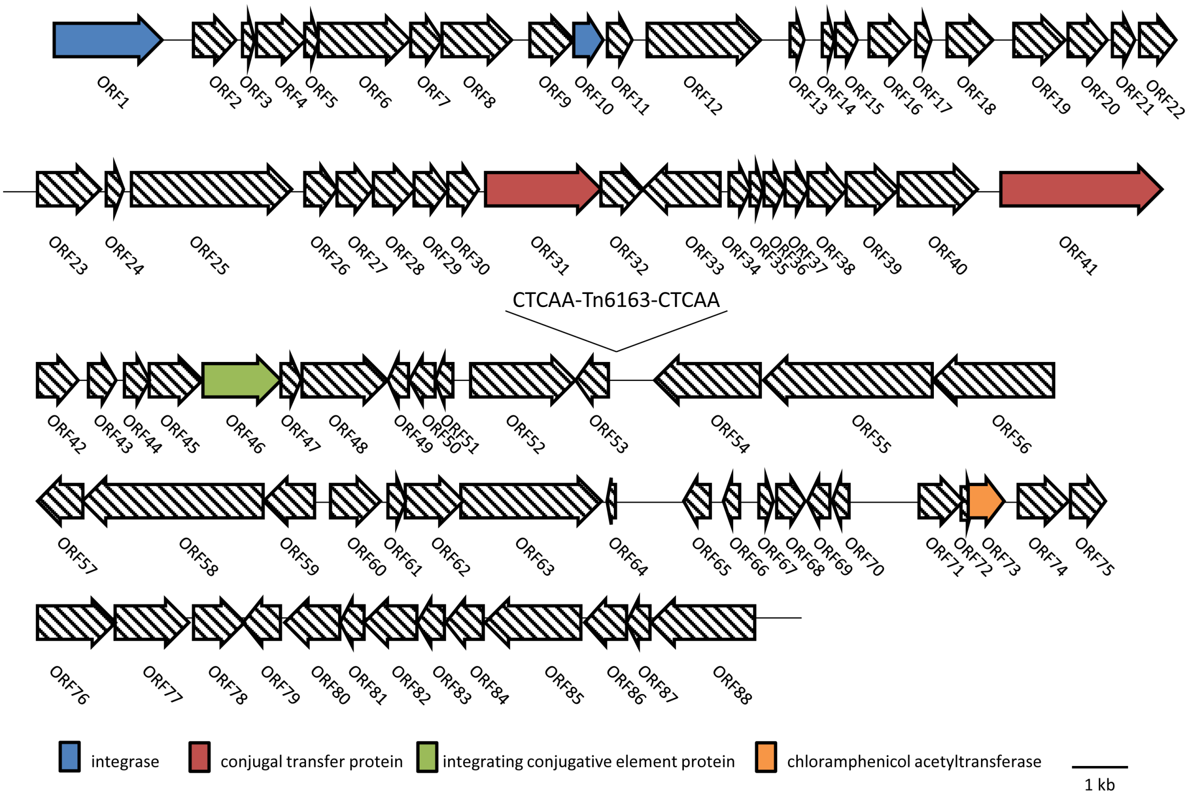


Figure S4: GI2 and insertion site of Tn*6163*.
